# Supplementary material for: Development of a Cell-Based SARS-CoV-2 Pseudovirus Neutralization Assay Using Imaging and Flow Cytometry Analysis
Source: Int J Mol Sci. 2023 Aug 2;24(15):12332. doi: 10.3390/ijms241512332 (PMC10418775; doi:10.3390/ijms241512332)
Supplement: Supplementary file 1 [file ijms-24-12332-s001.zip › ijms-2501002-supplementary figures.pdf]

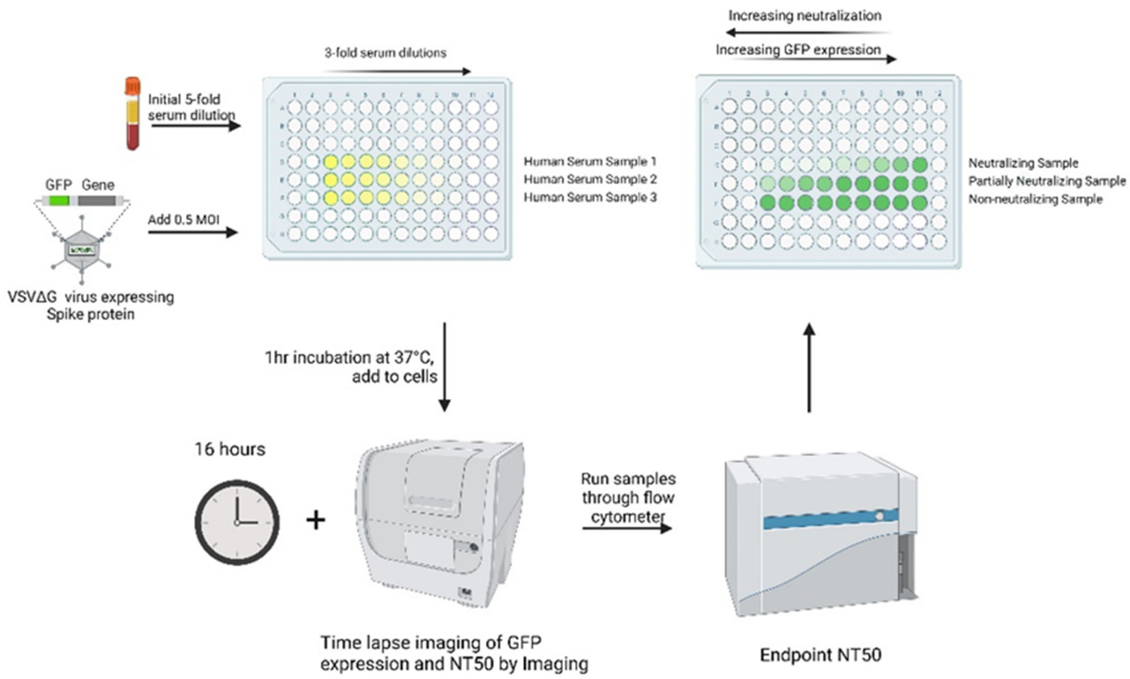

Figure S1. Protocol for Cell-Based Pseudovirus Neutralization Assay. Serum from naturally infected, vaccinated, or SARS-CoV-2 seronegative individuals was serially diluted 9 times. A VSVΔG expressing the Wuhan-Hu-1 Spike protein that contained a GFP reporter was added at a final MOI of 0.5. The serum and virus mixture was incubated for 1 hour at 37°C and then added to HEK293T-Ace2-TMPRSS2-mCherry cells. The plates were immediately placed on the Cytation 5 for time-lapse imaging for 16 hours. After imaging was finished, cells were processed on an NxT Attune Flow Cytometer. Percent GFP was determined either by MATLAB or by FlowJo for imaging or FC methods, respectively. Percent reduction was calculated by normalizing based on the virus and media control wells. NT50 values were determined for both methods and compared.

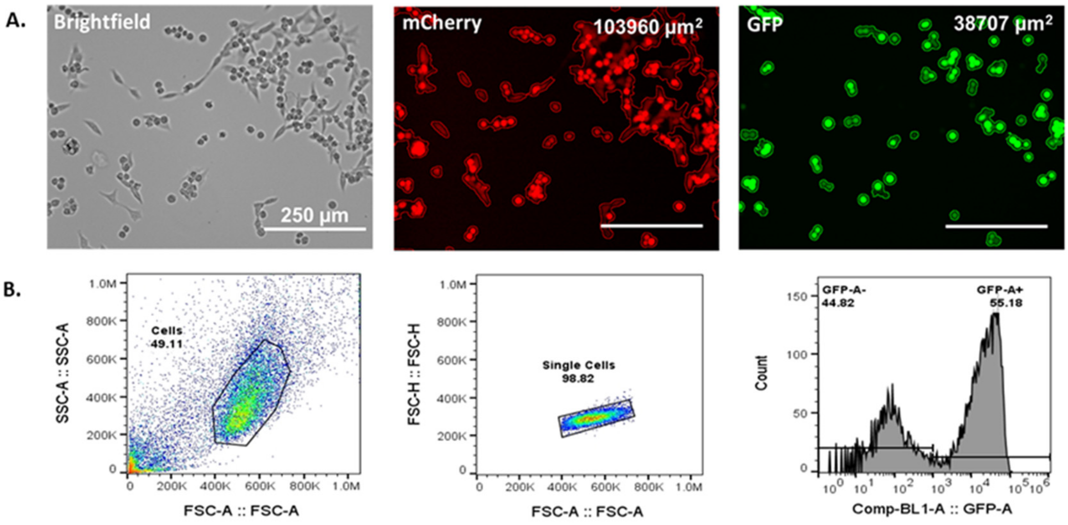

Figure S2: Segmentation for Imaging Method (A) and Gating Strategy for Flow Cytometry Method (B).
(A) Live cell imaging was performed with brightfield and fluorescence imaging. Fluorescence imaging used mCherry to identify all cells and green fluorescence protein (GFP) to identify pseudovirus infected cells. Automated image processing resulted in segmentation of total cells (red outline) and infected cells (green outline). Analysis enabled quantification of the percent infected cells as determined by cell area (37.2% infected). Above shows one out of nine fields of view of cells that were analyzed for each condition. The resulting metrics enabled quantification of infection

and serum neutralization in HEK293T-ACE2-TMPRSS2 target cells. (B) The gating strategy for flow cytometry analysis gated on the main population, single cells, and GFP positive cells. Only wells with at least 1500 single cells were included in the analysis.
