# Supplementary material for: Development of a Cell-Based SARS-CoV-2 Pseudovirus Neutralization Assay Using Imaging and Flow Cytometry Analysis
Source: Int J Mol Sci. 2023 Aug 2;24(15):12332. doi: 10.3390/ijms241512332 (PMC10418775; doi:10.3390/ijms241512332)
Supplement: Supplementary file 1 [file ijms-24-12332-s001.zip › MDPI-Supplemental Table S1.pdf]

Supplemental Table S1. Characteristics of Serum Samples Collected for Study.

**Sample Type**	**Sample Number**	**Year Collected**
Natural Infection	35	2020
Negative	28	Pre-2019
Vaccination	15	2021
